# Association between creatinine clearance and mortality in Chinese patients with osteoporotic fractures: a retrospective cohort study

**DOI:** 10.3389/fmed.2025.1550525

**Published:** 2025-08-14

**Authors:** Ming Su, Peng Zhou, Min-zhe Xu, Ya-qin Gong, Jian Jin, Wen-bin Hu, Ke Lu, Chong Li, Yi Yin

**Affiliations:** ^1^Department of Orthopedics, Affiliated Kunshan Hospital of Jiangsu University, Suzhou, Jiangsu, China; ^2^Kunshan Biomedical Big Data Innovation Application Laboratory, Suzhou, Jiangsu, China; ^3^Information Department, Affiliated Kunshan Hospital of Jiangsu University, Suzhou, Jiangsu, China; ^4^Kunshan Municipal Health and Family Planning Information Center, Suzhou, Jiangsu, China; ^5^Chronic Disease Department, Kunshan Center for Disease Control and Prevention, Suzhou, Jiangsu, China

**Keywords:** creatinine clearance, osteoporotic fractures, mortality, renal function, prognostic marker

## Abstract

**Background:**

Creatinine clearance (CCR) is a vital biomarker for evaluating renal function, indicating the efficiency of the kidneys in filtering blood waste. However, the link between CCR and mortality in hospitalized patients with osteoporotic fractures (OPFs) remains unclear. The increasing prevalence of OPFs in elderly populations, coupled with known complications of renal dysfunction, underscores the critical importance of understanding this relationship. This study aimed to investigate the association between CCR levels and mortality in a cohort of hospitalized patients with OPFs, with the goal of establishing evidence-based guidelines for risk stratification and management strategies.

**Methods:**

A retrospective cohort study analyzed data from 3,177 patients hospitalized with OPFs between 6 December 2018 and 31 December 2023. A multivariate Cox regression analysis was used to evaluate the relationship between CCR and mortality while adjusting for potential confounding variables, including laboratory parameters, clinical characteristics, and lifestyle factors. Subgroup analyses, smoothed curve fitting with threshold analyses, Kaplan–Meier curves, and sensitivity analyses were performed.

**Results:**

A linear correlation between CCR and mortality was observed, with each 1-point increment in CCR correlating with a 2% reduction in mortality risk (hazard ratio (HR) = 0.98; 95% confidence interval (CI): 0.97, 0.98; *p* < 0.01). Patients were categorized into three groups based on CCR: Group 1 (CCR ≤ 80 mL/min), Group 2 (80 < CCR ≤ 120 mL/min), and Group 3 (CCR > 120 mL/min). Group 2 exhibited a 51% lower hazard of mortality than Group 1 (HR = 0.49, 95% CI: 0.34, 0.71; *p* < 0.01), while Group 3 showed an 87% reduction in mortality risk (HR = 0.13, 95% CI: 0.05, 0.36; *p* < 0.01). Subgroup analyses confirmed the robustness of these findings even after adjusting for other covariates. Linear association was detected using smoothed curve fitting and threshold analysis. The Kaplan–Meier survival curves revealed a negative relationship between CCR levels and the cumulative mortality hazard. Sensitivity analyses demonstrated a stable direct association between CCR and the cumulative mortality hazard.

**Conclusion:**

This study demonstrated a significant association between CCR and mortality among hospitalized patients with OPFs, validating CCR as a valuable prognostic marker for assessing mortality risk.

## 1 Introduction

Osteoporotic fractures (OPFs) constitute a significant public health challenge, particularly among the elderly, due to their strong association with increased morbidity and mortality (1–3). These fractures are prevalent in aging populations and often result from weakened bones due to osteoporosis, which diminishes bone density and strength (4–7). Osteoporotic fractures frequently lead to prolonged hospital stay, reduced quality of life, and increased healthcare costs. The complications stemming from OPFs can be severe, including permanent disability, increased dependency, and a higher risk of subsequent fractures (8, 9). Moreover, these complications pose substantial socioeconomic burdens on the healthcare systems worldwide. Thus, understanding the factors that influence outcomes in patients with OPFs is crucial for developing effective management strategies to mitigate these adverse effects (10).

Among the various determinants of health outcomes, renal function is particularly vital because it plays a crucial role in numerous physiological processes (11). Creatinine clearance (CCR) serves as a key biomarker of renal performance, reflecting the capacity of the kidneys to filter waste products from the bloodstream (12, 13). Elevated serum creatinine levels often indicate compromised renal function, which has been linked to numerous complications, including cardiovascular issues, infections, and increased mortality rates (14–17). This increase is particularly concerning for elderly patients with OPFs who may have compromised renal function due to age-related decline. As renal function deteriorates, the risk of poor outcomes in patients with OPFs increases, making it essential to evaluate renal performance as part of the overall clinical assessment (18).

Despite the known importance of renal function in patient outcomes (19), the specific relationship between CCR and mortality in hospitalized patients with OPFs has not been thoroughly investigated. Although previous studies have examined various predictors of mortality in patients with OPFs (20–22), the prognostic value of CCR remains poorly understood. This knowledge gap underscores the urgent need for research exploring whether CCR can serve as a reliable prognostic marker for mortality in this vulnerable population. By analyzing a substantial cohort of patients, this study aimed to elucidate the correlation between CCR levels and mortality among patients diagnosed with OPFs. The findings from this investigation could provide invaluable insights into the role of renal function in determining patient prognosis and potentially inform clinical practices aimed at improving outcomes.

Our research hypothesis posits that the CCR level at admission may serve as an independent predictor of mortality in patients with OPFs. Ultimately, this study seeks to determine if CCR can be integrated into clinical management strategies to better identify high-risk patients and tailor interventions accordingly. Such an approach could significantly enhance patient care, reduce mortality, and improve the overall quality of life of individuals with osteoporotic fractures. By prioritizing renal health, healthcare providers can potentially mitigate the risks associated with OPFs, paving the way for more holistic and effective treatment frameworks.

## 2 Materials and methods

### 2.1 Data origin

We conducted a study using electronic medical records of patients aged 50 years and older residing in Kunshan, Jiangsu Province, China, who had recently been diagnosed with OPFs necessitating surgical hospitalization. These admitted patients were free of fractures for at least 5 years prior to their admission, resulting in their classification as first-time OPF cases. The fractures examined in this study were localized to the wrist, proximal humerus, hips, and vertebrae. These fractures were identified based on the 10th Revision of the International Statistical Classification of Diseases and Related Health Problems (ICD-10), using codes beginning with S22, S32, S42, S52, or S72. Specific ICD-10 codes were selected based on the established osteoporotic fracture classification criteria from previous epidemiological studies. In this study, patient clinical variables were systematically collected, including age, sex, weight, prothrombin time (PT), activated partial thromboplastin time (APTT), platelets, hemoglobin, albumin, calcium, neutrophils, lymphocytes, monocytes, potassium, uric acid, American Society of Anesthesiologists (ASA) category (1/2/≥3) (23), hypertension, diabetes, tumor, shock, and smoking. Laboratory parameters were obtained using standardized hospital protocols and analyzed in an accredited clinical laboratory. All clinical indicators were evaluated within 3 days of admission. This study was structured as a retrospective cohort study, encompassing patient records collected from December 2018 to December 2023. The study protocol was approved by the Institutional Review Board of Kunshan Hospital (approval no. 2024–03-053-H00-K01).

The study specifically enrolled patients with initial OPFs who were admitted consecutively to Kunshan Hospital affiliated with Jiangsu University. A consecutive enrollment approach was adopted to minimize selection bias.

### 2.2 Ethical statement

The study was approved by the Ethics Committee of the Affiliated Kunshan Hospital of Jiangsu University, Suzhou, China (approval no. 2024–03-053-H00-K01) and adhered to the principles outlined in the Declaration of Helsinki. All patient data were anonymized and de-identified prior to the analysis to ensure confidentiality and unbiased investigation. All patients provided written informed consent before participating in the study.

### 2.3 Study design and patient clinical cohorts

**Figure 1:**
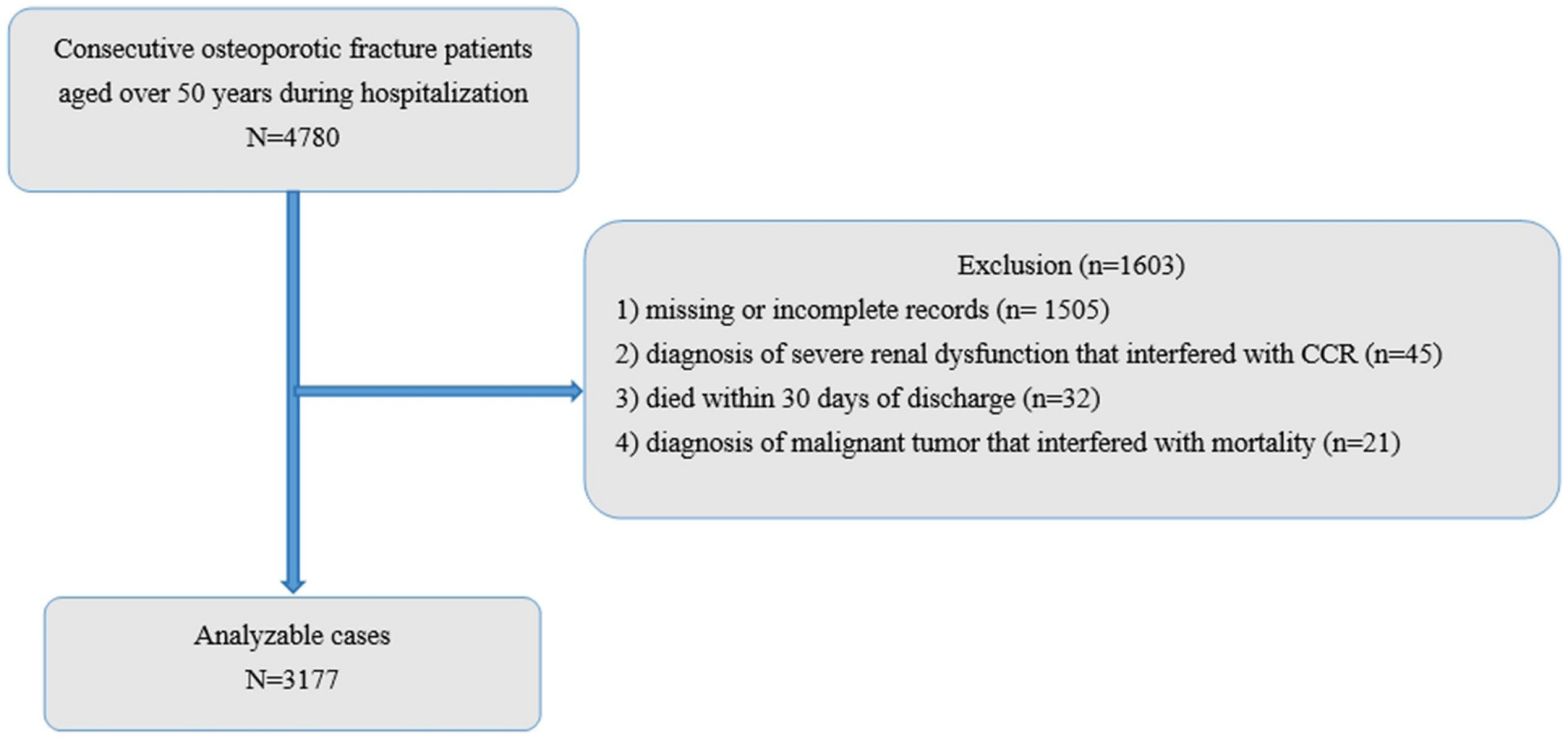
Schematic representation of the study design. CCR, creatinine clearance.

This retrospective observational study enrolled patients from 6 December 2018 and 31 December 2023. Data on participation in this study were available throughout the study. Follow-up evaluations were performed for at least 30 days. The study enrolled 4,780 sequentially admitted patients aged ≥ 50 years who were newly diagnosed with significant OPFs. Among these participants, 1,603 were excluded based on the predefined criteria (Figure 1). The exclusion criteria were as follows: (1) missing or incomplete records (*n* = 1,505), (2) diagnosis of severe renal dysfunction that interfered with CCR (*n* = 45) (24), (3) death within 30 days of discharge (*n* = 32) (25), and (4) diagnosis of malignant tumor that interfered with mortality (*n* = 21) (24).

### 2.4 Exposure and outcome variables

CCR is determined with the Cockcroft-Gault formula: (140-age[year]) × body weight [kg]/ plasma creatinine [mg/dl] × 72 (×0.85 if female) (26, 27). This calculated CCR value served as our primary exposure variable (28). Patients with CCR < 80 mL/min were classified into the decreased group, those with CCR between 80 and 120 mL/min were categorized into the normal group, and those with CCR > 120 mL/min were classified into the elevated group (29). The primary outcome measure was mortality among surgically treated OPF patients hospitalized in the Kunshan region. The follow-up period extended from the initial hospital discharge date to either the date of death or study conclusion (31 December 2023), with mortality serving as the primary endpoint.

### 2.5 Covariate variables

In this study, we assessed and recorded covariate variables including PT, APTT, platelets, hemoglobin, albumin, calcium, neutrophils, lymphocytes, monocytes, potassium, uric acid, ASA category (1/2/≥3), hypertension, diabetes, tumor, shock, and smoking. Laboratory measurements were conducted using standardized methods and equipment. PT and APTT were performed using the CN-6000 automated coagulation analyzer (Sysmex Corporation, Kobe, Japan) through the coagulation method. Platelet counts were determined using flow cytometry with impedance on a Sysmex XN-10 (B4) hematology analyzer (Sysmex Corporation, Kobe, Japan). Hemoglobin levels were assessed using the Sysmex XN-10 hematology analyzer (Sysmex Corporation, Kobe, Japan) using the sodium lauryl sulfate-hemoglobin (SLS-Hb) method, and albumin levels were quantified using the Beckman AU5800 automated biochemistry analyzer (Beckman Coulter, Brea, CA, USA), using the Biuret method. The Sysmex XN-10 hematology analyzer (Sysmex Corporation, Kobe, Japan), utilizing flow cytometry with nuclear staining, was used to measure neutrophil, lymphocyte, and monocyte counts. Potassium levels were assessed using a Beckman AU5800 automated biochemistry analyzer (Beckman Coulter, Brea, CA, USA) using the ion-selective electrode (ISE) method. Uric acid levels were determined with a Beckman AU5800 automated biochemistry analyzer (Beckman Coulter, Brea, CA, USA) using the uricase-peroxidase method. For clinical assessment, the ASA category was assigned according to the anesthesiologist’s evaluation of the patient’s health status prior to surgery. The ASA classification system categorizes patients based on their preoperative physical status and potential anesthesia-related risks (23).

### 2.6 Statistical analyses

**Figure 2:**
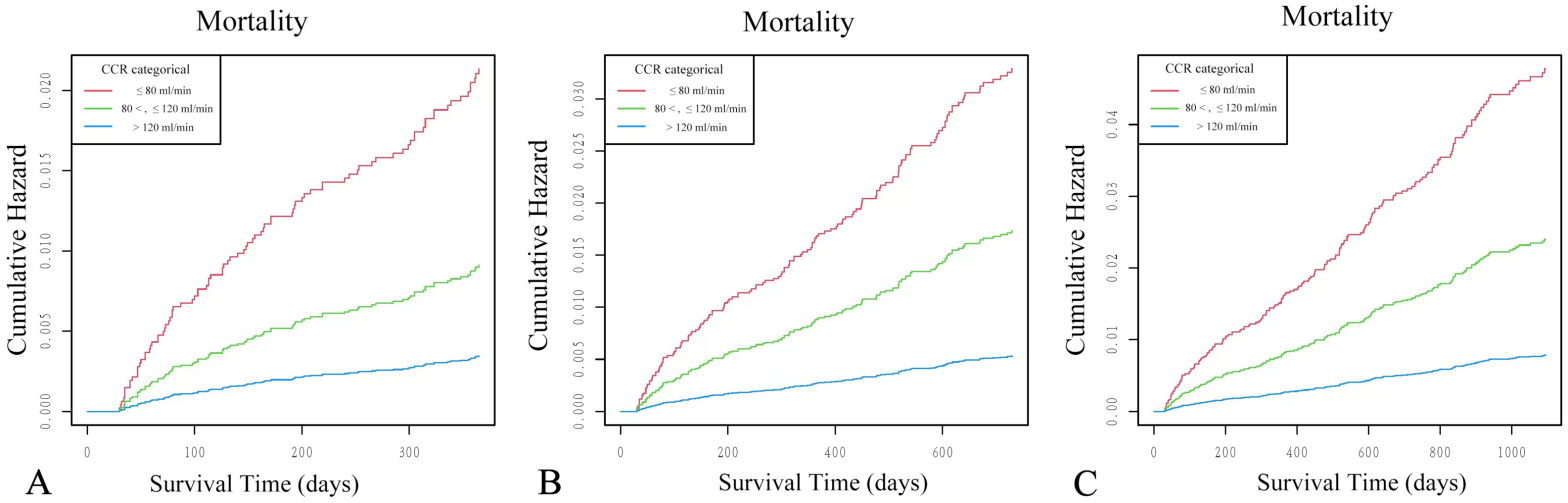
The Kaplan–Meier curves of varying sensitivity analyses to estimate the cumulative hazard of mortality among patients stratified by CCR levels: Group 1 (CCR ≤ 80 mL/min) (red line), Group 2 (80 < CCR ≤ 120 mL/min) (green line), and Group 3 (CCR > 120 mL/min) (blue line). Analyses were conducted with follow-up periods censored at 1 **(A)**, 2 **(B)**, and 3 **(C)** years. All curves were adjusted for PT, APTT, platelet, hemoglobin, albumin, calcium, neutrophils, lymphocytes, monocytes, potassium, uric acid, ASA category, hypertension, diabetes, tumor, shock, and smoking.

The study cohort was stratified into three groups according to the CCR levels. Categorical variables are presented as counts and percentages, and continuous variables are expressed as mean ± standard deviation (SD). Non-normally distributed data were analyzed using the Mann–Whitney *U*-tests, whereas independent two-tailed *t*-tests were used to compare data that followed a normal distribution. The Chi-square tests were used to evaluate the differences in the categorical data, which were reported as counts and percentages. If the chi-square test assumptions were not satisfied, the Fisher’s exact test was used as an alternative. Individuals were classified into three categories: Group 1, Group 2, and Group 3. To assess the relationship between various CCR levels and mortality, we applied Cox proportional hazards regression models while adjusting for covariates. Initially, collinearity assessments were conducted using variance inflation factor (VIF) evaluations. The need for adjusting covariates was subsequently assessed based on the following criteria: Criterion 1 involved either adding a confounding variable to the foundational model (which initially encompassed only the CCR (0 < CCR < 300 mL/min) and mortality without any additional variables) or excluding it from the comprehensive model. This adjustment sought to achieve a minimum alteration of 10% in the adjusted odds ratio (OR). Criterion 2 involved either satisfying the conditions of the first criterion or identifying a covariate with a *p*-value of less than 0.1 in the univariate analysis. No adjustments were made to the initial model. However, Model 2 was modified to account for PT, APTT, platelet count, hemoglobin, albumin, calcium, neutrophils, lymphocytes, monocytes, and potassium. In contrast to Model 2, Model 3 incorporated further modifications, which were determined by either satisfying the first or second criteria. In Model 3, modifications were made to account for factors including PT, APTT, platelet, hemoglobin, albumin, calcium, neutrophil, lymphocyte, monocyte, potassium, uric acid, ASA category, hypertension, diabetes, tumor, shock, and smoking. The reliability of the research and differences among patient groups were assessed using subgroup analyses in which patients were categorized based on particular variables. A smoothed line was used to assess both linear and non-linear correlations. We also used the Kaplan–Meier curves to determine the cumulative hazard rates for mortality. Subsequently, in the sensitivity analysis, follow-up periods of 1, 2, and 3 years were used to evaluate whether potential differences in the follow-up duration affected the three groups (Figure 2; Table 1).

**Table 1:** Sensitivity analyses association between CCR and mortality using different censor strategies.

Exposure	Model 1^a^ *N* = 3,177 HR (95% CI) *p*-value	Model 2^b^ *N* = 3,088 HR (95% CI) *p*-value	Model 3^c^ *N* = 2,946 HR (95% CI) *p*-value
Censored at 1 year			
CCR categorical			
≤80 (ml/min)	Reference	Reference	Reference
>80, ≤120 (ml/min)	0.30 (0.17, 0.53) < 0.01	0.30 (0.17, 0.54) < 0.01	0.45 (0.24, 0.85) 0.01
>120 (ml/min)	0.11 (0.03, 0.44) < 0.01	0.11 (0.03, 0.44) < 0.01	0.19 (0.04, 0.78) 0.02
CCR continuous (ml/min)	0.97 (0.96, 0.98) < 0.01	0.97 (0.96, 0.98) < 0.01	0.98 (0.97, 0.99) < 0.01
Censored at 2 years			
CCR categorical			
≤80 (ml/min)	Reference	Reference	Reference
>80, ≤120 (ml/min)	0.35 (0.23, 0.54) < 0.01	0.35 (0.22, 0.54) < 0.01	0.56 (0.35, 0.89) 0.01
>120 (ml/min)	0.10 (0.03, 0.32) < 0.01	0.10 (0.03, 0.32) < 0.01	0.19 (0.06, 0.60) < 0.01
CCR continuous (ml/min)	0.97 (0.97, 0.98) < 0.01	0.97 (0.96, 0.98) < 0.01	0.98 (0.97, 0.99) < 0.01
Censored at 3 years			
CCR categorical			
≤80 (ml/min)	Reference	Reference	Reference
>80, ≤120 (ml/min)	0.35 (0.24, 0.52) < 0.01	0.35 (0.24, 0.52) < 0.01	0.52 (0.35, 0.79) < 0.01
>120 (ml/min)	0.11 (0.04, 0.30) < 0.01	0.11 (0.04, 0.30) < 0.01	0.18 (0.07, 0.50) < 0.01
CCR continuous (ml/min)	0.97 (0.97, 0.98) < 0.01	0.97 (0.97, 0.98) < 0.01	0.98 (0.97, 0.99) < 0.01

a No adjustment.

b Adjusted for PT, APTT, platelet, hemoglobin, albumin, calcium, neutrophils, lymphocytes, monocytes, and potassium.

c Adjusted for PT, APTT, platelet, hemoglobin, albumin, calcium, neutrophils, lymphocytes, monocytes, potassium, uric acid, ASA category, hypertension, diabetes, tumor, shock, and smoking.

Statistical analyses were performed using Empower Stats (Empowerstats.com, X&Y Solutions, Inc., Boston, MA, USA). The significance threshold was set at a *p*-value of 0.05 or below.

## 3 Results

### 3.1 Baseline study population characteristics

This retrospective study included 3,177 patients who met the inclusion criteria. Table 2 summarizes the baseline characteristics of the patients, of whom 31.35% (*n* = 996) were male and 68.65% (*n* = 2,181) were female, with a mean age of 67.90 ± 10.98 years. The study cohort exhibited a mean CCR value of 81.28 ± 35.83 mL/min. Based on the CCR values, we classified the patients into three categories, which revealed significant differences in gender and age between these groups. Gender-specific analysis revealed that a higher proportion of female patients with lower CCR levels were significantly older. Detailed analyses of covariates across the different CCR categories are presented in Table 2.

**Table 2:** Characteristic of study participants.

Characteristics	Total Mean ± SD	CCR categorical (ml/min)			*p*-value*
		≤80 Mean ± SD	>80, ≤120 Mean ± SD	>120 Mean ± SD	
*N*	3,177	1,695	1,064	418	
Gender, *N* (%)					<0.01
Female	2,181 (68.65%)	1,383 (81.59%)	625 (58.74%)	173 (41.39%)	
Male	996 (31.35%)	312 (18.41%)	439 (41.26%)	245 (58.61%)	
Age, years	67.90 ± 10.98	72.92 ± 10.45	63.29 ± 8.60	59.27 ± 7.38	<0.01
CCR, ml/min	81.28 ± 35.83				
PT, s	11.86 ± 1.50	11.86 ± 1.61	11.89 ± 1.29	11.82 ± 1.55	0.01
APTT, s	28.24 ± 4.29	28.70 ± 4.46	27.77 ± 3.77	27.58 ± 4.61	<0.01
Platelet, ×10^9^/L	178.69 ± 63.58	171.21 ± 61.52	185.52 ± 65.15	191.66 ± 63.87	<0.01
Hemoglobin, g/L	123.68 ± 18.27	122.44 ± 19.60	125.83 ± 16.48	123.23 ± 16.44	<0.01
Albumin, g/L	39.69 ± 4.42	39.34 ± 4.61	40.10 ± 4.13	40.03 ± 4.22	<0.01
Calcium, mmol/L	2.19 ± 0.13	2.19 ± 0.13	2.19 ± 0.13	2.17 ± 0.13	0.05
Neutrophil, ×10^9^/L	6.76 ± 3.21	6.66 ± 3.21	6.88 ± 3.23	6.88 ± 3.14	0.12
Lymphocyte, ×10^9^/L	1.21 ± 0.53	1.21 ± 0.54	1.21 ± 0.51	1.21 ± 0.55	0.97
Monocyte, ×10^9^/L	0.52 ± 0.31	0.52 ± 0.26	0.52 ± 0.40	0.51 ± 0.22	0.69
Potassium, mmol/L	3.85 ± 0.44	3.91 ± 0.46	3.80 ± 0.41	3.70 ± 0.39	<0.01
Uric acid, μmol/L	281.19 ± 91.47	297.75 ± 96.47	269.82 ± 82.80	243.06 ± 74.47	<0.01
ASA, *N* (%)					<0.01
1	467 (14.70%)	202 (11.92%)	184 (17.29%)	81 (19.38%)	
2	2083 (65.56%)	1,014 (59.82%)	763 (71.71%)	306 (73.20%)	
≥3	627 (19.74%)	479 (28.26%)	117 (11.00%)	31 (7.42%)	
Hypertension, *N* (%)					<0.01
No	2,787 (87.72%)	1,429 (84.31%)	971 (91.26%)	387 (92.58%)	
Yes	390 (12.28%)	266 (15.69%)	93 (8.74%)	31 (7.42%)	
Diabetes, *N* (%)					0.05
No	3,069 (96.60%)	1,625 (95.87%)	1,037 (97.46%)	407 (97.37%)	
Yes	108 (3.40%)	70 (4.13%)	27 (2.54%)	11 (2.63%)	
Tumor, *N* (%)					0.40
No	3,143 (98.93%)	1,673 (98.70%)	1,055 (99.15%)	415 (99.28%)	
Yes	34 (1.07%)	22 (1.30%)	9 (0.85%)	3 (0.72%)	
Shock, *N* (%)					0.64
No	3,175 (99.94%)	1,693 (99.88%)	1,064 (100.00%)	418 (100.00%)	
Yes	2 (0.06%)	2 (0.12%)	0 (0.00%)	0 (0.00%)	
Smoking, *N* (%)					<0.01
No	2,769 (91.63%)	1,571 (96.56%)	881 (88.54%)	317 (79.25%)	
Yes	253 (8.37%)	56 (3.44%)	114 (11.46%)	83 (20.75%)	

CCR, creatinine clearance; SD, standard deviation; PT, Prothrombin Time; APTT, Activated Partial Thromboplastin Time; ASA, American Society of Anesthesiologists.

### 3.2 Association between CCR and mortality

Three models were used in the subsequent phase to analyze the correlation between CCR and mortality in participants with OPFs (Table 3). In unadjusted Model 1, we observed a significant negative correlation between CCR and mortality (HR = 0.97, 95% CI: 0.97 to 0.98, *p* < 0.01). After adjusting for laboratory parameters such as PT, APTT, platelet count, hemoglobin, albumin, calcium, neutrophils, lymphocytes, monocytes, and potassium in Model 2, the observed relationships remained consistent. Specifically, CCR showed a significant negative association with mortality (HR = 0.97, 95% CI: 0.97 to 0.98, *p* < 0.01). Model 3 further included adjustments for clinical characteristics, including uric acid, ASA, hypertension, diabetes, tumor, shock, and smoking, and consistently showed a negative correlation. To facilitate clinical interpretation, the CCR levels were categorized into three groups based on the normal range of 80–120 mL/min. Group 1 included patients with a clearance rate of ≤ 80 mL/min, Group 2 included those with a clearance rate of > 80 and ≤ 120 mL/min, and Group 3 included patients with a clearance rate of > 120 mL/min. The survival analysis indicated that, in Model 1, the mortality rate in Group 2 was 64% lower than that in Group 1, whereas Group 3 exhibited a 91% reduction in mortality rate relative to Group 1 (Table 3). These significant survival benefits remained consistent across Models 2 and 3 after adjusting for confounding factors.

**Table 3:** Association between CCR and mortality using Cox regression analysis in different models.

Exposure	Model 1^a^ *N* = 3,177 HR (95% CI) *p*-value	Model 2^b^ *N* = 3,088 HR (95% CI) *p*-value	Model 3^c^ *N* = 2,946 HR (95% CI) *p*-value
CCR categorical			
Group 1 (≤80 ml/min)	Reference	Reference	Reference
Group 2 (>80, ≤120 ml/min)	0.36 (0.25, 0.51) < 0.01	0.34 (0.24, 0.49) < 0.01	0.49 (0.34, 0.71) < 0.01
Group 3 (>120 mL/min)	0.09 (0.03, 0.25) < 0.01	0.09 (0.03, 0.23) < 0.01	0.13 (0.05, 0.36) < 0.01
CCR continuous (ml/min)	0.97 (0.97, 0.98) < 0.01	0.97 (0.97, 0.98) < 0.01	0.98 (0.97, 0.98) < 0.01

a No adjustment.

b Adjusted for PT, APTT, platelet counts, hemoglobin, albumin, calcium, neutrophils, lymphocytes, monocytes, and potassium.

c Adjusted for PT, APTT, platelet, hemoglobin, albumin, calcium, neutrophil, lymphocyte, monocyte, potassium, uric acid, ASA category, hypertension, diabetes, tumor, shock, and smoking.

### 3.3 Subgroup analyses

To evaluate the robustness of our findings, we performed comprehensive subgroup analyses, stratified by laboratory parameters (PT, APTT, platelet count, hemoglobin, albumin, calcium, and blood cell counts), clinical characteristics (hypertension, diabetes, and malignancy), and lifestyle factors (smoking status), to validate that the association between CCR and mortality remained significant after controlling for potential confounders in the fully adjusted multivariate Cox regression model. For each subgroup analysis, we adjusted for covariates that were not used in stratification. The studies showed consistent patterns in their results, with no detected interactions due to stratification (Supplementary Table 1).

### 3.4 Spline smoothing plot and threshold analysis

**Figure 3:**
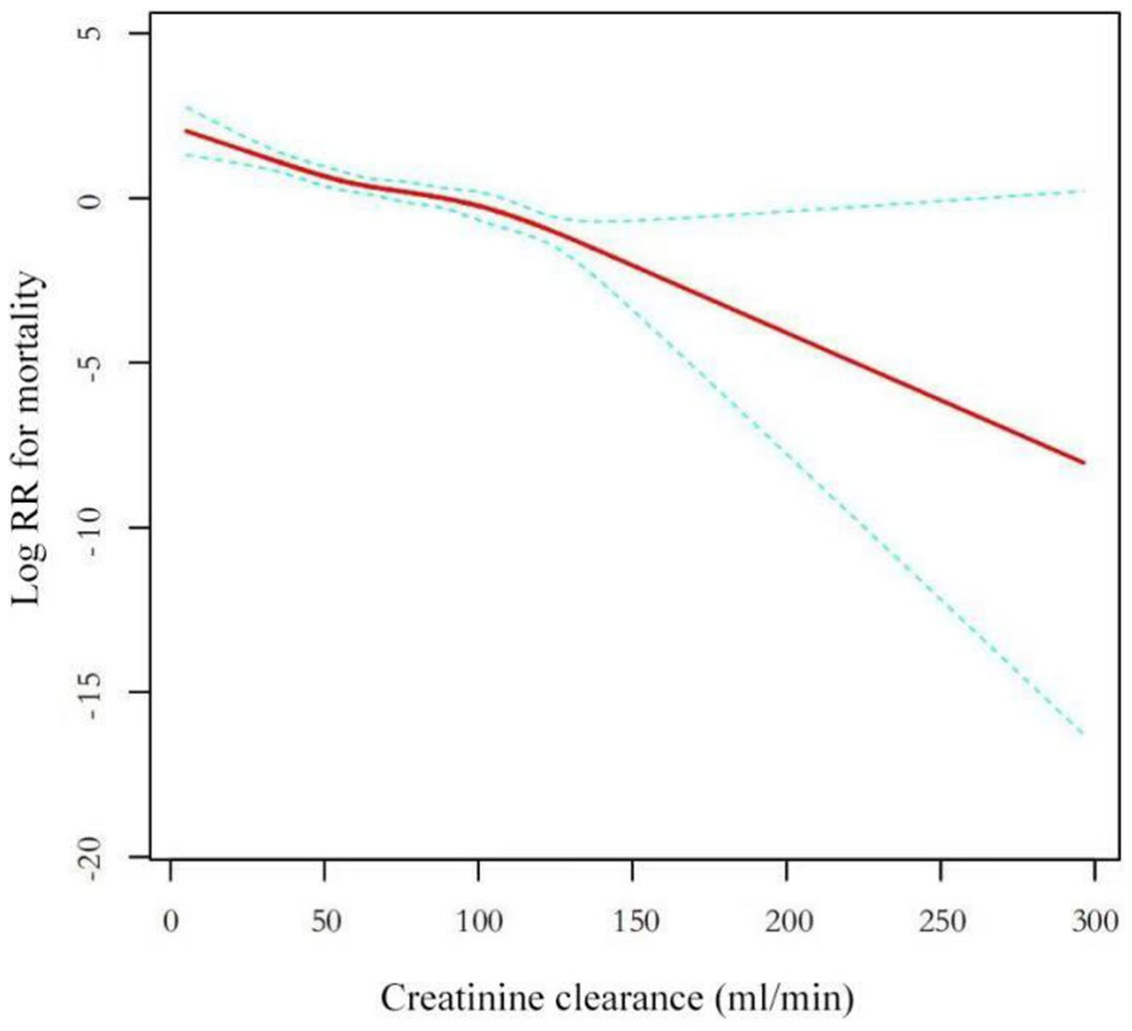
Adjusted smoothed curves corresponding to the relationship between the CCR and mortality among inpatients with OPFs. The red curve in the middle represents the estimated value, and the blue curves on either side represent the 95% CI. The adjusted factors were PT, APTT, platelet, hemoglobin, albumin, calcium, neutrophils, lymphocytes, monocytes, potassium, uric acid, ASA category, hypertension, diabetes, tumor, shock, and smoking. CCR, creatinine clearance; OPFs, osteoporotic fractures.

In hospitalized patients with OPFs, we observed a negative linear association between CCR and mortality after adjusting for confounding factors, including PT, APTT, platelet count, hemoglobin, albumin, calcium, neutrophils, lymphocytes, monocytes, potassium, uric acid, ASA category, hypertension, diabetes, tumor, shock, and smoking (Figure 3). The threshold effect analysis using Model 3, which examined the relationship between CCR and mortality, is presented in Table 4. The analysis revealed a linear correlation between CCR levels and mortality among inpatients with OPFs, as evidenced by the *p*-value obtained from the logarithmic likelihood ratio test (LRT) = 0.39. The analysis demonstrated a negative association with mortality (HR = 0.98; 95% CI: 0.97, 0.98; *p*-value<0.01). These findings indicate that, for every 1-point increase in CCR, there was a corresponding 2% decrease in mortality.

**Table 4:** Threshold analyses examining the relationship between CCR and mortality.

	Model 3^a^ Mortality HR (95% CI) *p*-value
Model A^b^	
One line slope	0.98 (0.97, 0.98) < 0.01
Model B^c^	
CCR turning point (K)	115.29
< K	0.98 (0.97, 0.99) < 0.01
> K	0.96 (0.91, 1.01) 0.11
Slope 2-Slope 1	0.98 (0.93, 1.03) 0.45
LRT^d^	0.39

a Adjusted for PT, APTT, platelet, hemoglobin, albumin, calcium, neutrophils, lymphocytes, monocytes, potassium, uric acid, ASA category, hypertension, diabetes, tumor, shock, and smoking.

b Linear analysis, *p*-value<0.05 indicates a linear relationship.

c Nonlinear analysis.

d *p*-value>0.05, indicating that Model B was insignificantly different from Model A, which indicates a linear relationship.

### 3.5 Analysis of the Kaplan–Meier survival curves based on CCR levels

**Figure 4:**
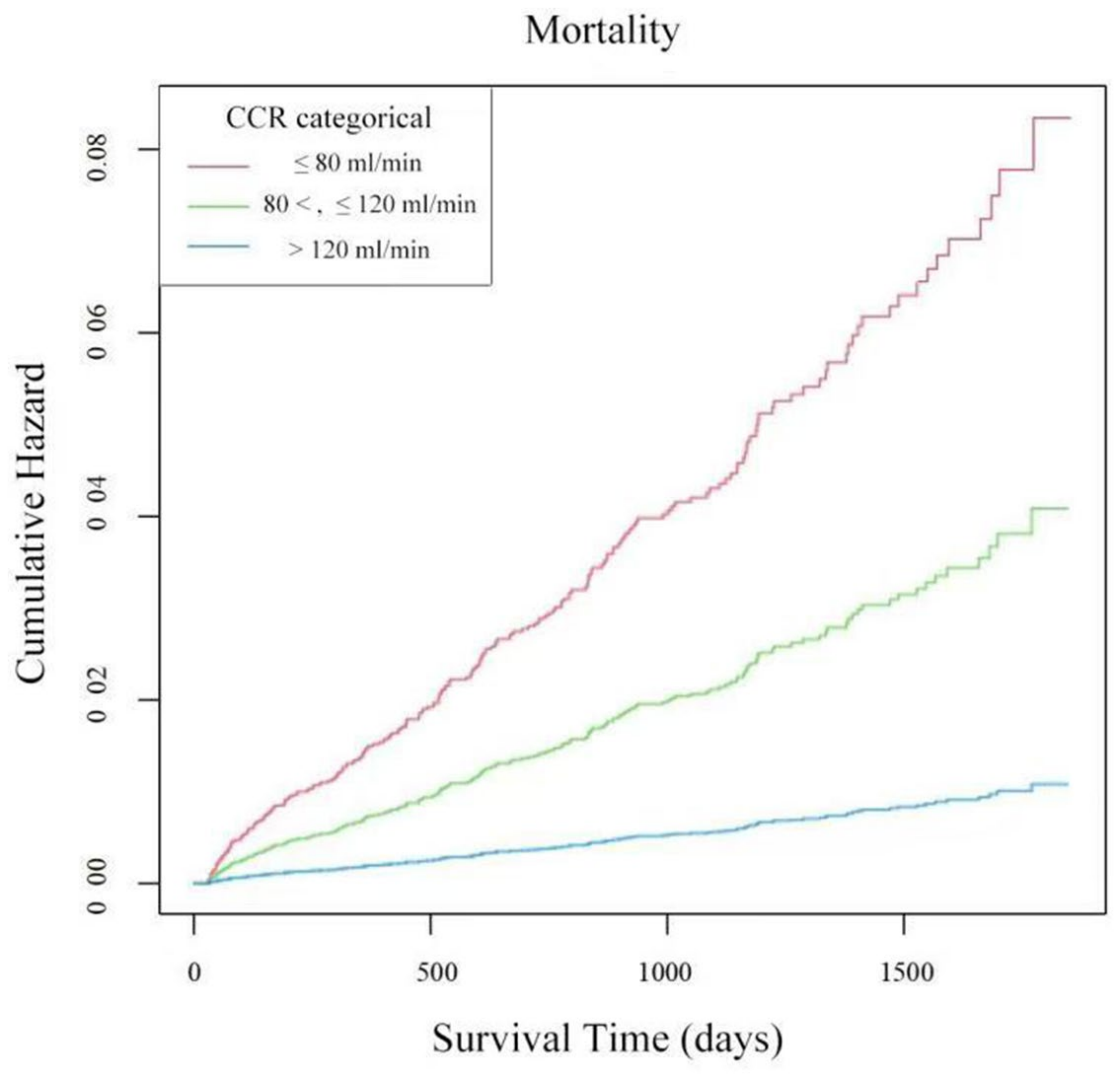
The Kaplan–Meier curves were used to estimate the cumulative hazard of mortality of patients in Group 1 (CCR ≤ 80 mL/min) (red line), Group 2 (80 < CCR ≤ 120 mL/min) (green line), and Group 3 (CCR > 120 mL/min) (blue line). The adjusted factors were PT, APTT, platelet, hemoglobin, albumin, calcium, neutrophils, lymphocytes, monocytes, potassium, uric acid, ASA category, hypertension, diabetes, tumor, shock, and smoking.

The patient cohort was stratified into three groups according to CCR: Group 1, Group 2, and Group 3. The relationship between different CCR groups and cumulative mortality hazard was evaluated using the Kaplan–Meier curves (Figure 4). We found that Group 3 and Group 2 exhibited a significantly lower cumulative hazard of mortality than Group 1 (all *p*-values <0.01). These results demonstrate that higher CCR levels were associated with a decreased mortality risk in our patient cohort.

### 3.6 Sensitivity analyses

Sensitivity analyses using 1-, 2-, and 3-year follow-up periods were conducted to evaluate the potential impact of varying follow-up durations on the association between the cumulative mortality hazard and CCR levels. The findings indicated that the association between CCR and the cumulative mortality hazard maintained a consistent pattern (Figure 2; Table 1).

## 4 Discussion

This study investigated the relationship between CCR and mortality in hospitalized patients diagnosed with OPFs. In this study, female patients comprised 68.65% of the total sample. It is believed that a combination of factors, including hormone levels, bone mineral density, age, lifestyle, and genetic predisposition, has contributed to the higher prevalence of osteoporotic fractures among women (30–33). Initially, a clear linear correlation was established between CCR and mortality. Through a rigorous statistical analysis, our findings demonstrated that, for every increment of 1 point in the CCR, there was a corresponding 2% decrease in the likelihood of mortality. Furthermore, when participants were categorized based on their CCR levels, patients with normal CCR (Group 2) exhibited a 51% decreased hazard of mortality compared to those with decreasing CCR (Group 1). Meanwhile, patients with increased CCR (Group 3) had an 87% lower mortality hazard than those in Group 1 (Table 3). These results indicate that CCR can potentially serve as an indicator of mortality among hospitalized patients with OPFs and that low CCR levels represent a modifiable risk factor that contributes to the increased mortality risk in this group002E.

CCR is an indicator of renal function and, in many instances, can represent the functionality of eGFR, serving as a crucial determinant of mortality and adverse outcomes across various clinical conditions (34, 35), particularly in patients with compromised kidney function and chronic diseases such as hypertension, dyslipidemia, diabetes, and vascular disease (36–38). The clinical significance of CCR lies in its comprehensive assessment of renal function and its proven value in disease prognosis. This index is derived from measurements of the patient’s age, sex, weight, and plasma creatinine levels, which enhances its diagnostic accuracy and increases its effectiveness in clinical settings (27, 39, 40).

Some studies have examined the association between CCR and mortality risk or disease activity in systemic lupus erythematosus (SLE) and colorectal cancer patients, which have been confirmed as important prognostic indicators across different illnesses. In a multicenter retrospective cohort study, Jiahuan Ge et al. identified significant correlations between declining CCR and mortality in SLE patients, particularly in relation to infections and renal insufficiency (41). In a cohort study, Chen et al. demonstrated that the incidence of postoperative complications (POCs) was significantly higher in patients with low CCR (28). Furthermore, Sakai S et al. demonstrated through their analysis of data from the Hirosaki University Graduate School of Medicine that low CCR is a significant risk factor for severe cardioembolic stroke (CES) in Japanese female patients. Their findings underscore the importance of monitoring CCR in clinical practice to identify high-risk individuals early and implement appropriate preventive measures, thereby improving patient outcomes (42). Moreover, low levels of CCR were identified as a modifiable risk factor that contributes to the heightened mortality risk in this population. These results align with existing research, reinforcing the significance of CCR in assessing patient outcomes (28, 41, 43). Based on previous evidence, low levels of CCR are considered a significant risk factor for the incidence or mortality of various diseases such as SLE, POC, and CES. The present study primarily investigated the effect of CCR levels on mortality in populations with OPFs. An analysis of hospitalized patients with primary OPFs from Kunshan Hospital, affiliated with Jiangsu University, reached similar conclusions. The results showed that CCR may serve as a prognostic marker for mortality in hospitalized patients with OPFs (HR = 0.98, *p*-value < 0.01). We found a significant association between CCR and mortality in hospitalized patients with OPFs. Specifically, patients with normal CCR face a mortality risk that is 0.49 times lower than those with decreasing CCR (95% CI: 0.34, 0.71). Additionally, those with increased CCR had a mortality risk that was 0.13 times lower than that of patients with decreasing CCR (95% CI: 0.05, 0.36) (Table 3).

Creatinine serves as a vital biomarker for assessing renal function, with elevated serum creatinine levels often indicating compromised kidney performance (44). Specifically, CCR is widely used as a more accurate indicator of the glomerular filtration rate and overall kidney function. Renal insufficiency is associated with various complications, which increase the risk of mortality (45). Increasing evidence indicates that reduced CCR levels are linked to a heightened risk of osteoporosis (46). Recent studies have consistently demonstrated that this association may be attributed to altered mineral metabolism and hormonal imbalances that occur during renal dysfunction. These findings underscore the relationship between kidney function and bone metabolism. Impaired renal function can negatively affect bone health through various biological pathways. In the context of chronic kidney disease (CKD), for instance, the decline in renal function results in disturbances in calcium and phosphate homeostasis and secondary hyperparathyroidism, which can accelerate bone loss and elevate fracture risk (47). CCR, serving as an indicator of glomerular filtration and overall renal health, may represent these pathogenic processes. A lower CCR may indicate diminished renal clearance of phosphorus and reduced activation of vitamin D, leading to increased levels of parathyroid hormone (PTH) and subsequent bone resorption (48, 49). More specifically, creatinine is a key component of CCR, which may explain the observed link between CCR and mortality in individuals with OPFs. These findings suggest a complex interplay between renal and bone metabolism. Furthermore, the relationship between CCR and OPFs could be bidirectional because decreased mobility following fractures may also affect kidney function. This bidirectional relationship warrants further investigation to fully understand its underlying mechanisms.

In addition to the impact of renal function indicators on the prognosis of OPFs, we also believe it is important to further explore the significance of CCR in evaluating mortality risk associated with common diseases in the elderly population. Our previous findings have demonstrated that CCR levels are significantly associated with mortality risk in patients with OPFs, suggesting that CCR could serve as an independent prognostic indicator. In fact, as a key marker of renal function, CCR also has important value in the prognosis assessment of other illnesses among older adults. Studies have shown that common diseases in the elderly patients include osteoporosis, arthritis, cardiovascular diseases, and neurological disorders (1, 11, 36, 45). These conditions not only significantly affect quality of life but also often coexist, resulting in more severe clinical consequences. For example, osteoporosis can lead to fractures, which may in turn increase the risk of cardiovascular diseases. Moreover, elderly patients with chronic kidney disease typically have a worse prognosis and are more likely to experience complications such as infections, osteoporosis, and fractures (24, 45). Given these considerations, we suggest that future research should further investigate the application of CCR in evaluating mortality risk related to common diseases in the elderly. A more in-depth analysis of the association between CCR and the prognosis of various geriatric diseases could help to clarify the clinical implications of our findings for geriatric practice and provide a reference for optimizing comprehensive management strategies in the elderly population.

Many existing studies have emphasized the importance of assessing patients’ CCR to improve clinical outcomes, as this evaluation can provide critical insights into renal function, guide appropriate medication dosing, and facilitate timely interventions that enhance patient care and recovery (50). In addition, related studies have suggested implementing multiple assessment methods to optimize the monitoring and management of renal function to improve patient treatment outcomes (51). These methods may include, but are not limited to, CCR measurement, estimated GFR calculations, and biomarker analysis; therefore, CCR should be considered as a primary parameter in clinical evaluations to more accurately monitor the renal function status of these patients. This recommendation is particularly pertinent for patients with comorbidities or those receiving nephrotoxic medications. In summary, CCR is a vital biomarker for renal function assessment, offering enhanced diagnostic accuracy over serum creatinine, particularly in at-risk populations. Its superiority for detecting subtle changes in renal function makes it an invaluable tool for early interventions. It serves as a significant prognostic indicator for mortality, enabling the early identification of high-risk individuals and guiding therapeutic interventions, thereby optimizing treatment strategies and improving patient outcomes.

Our findings revealed a notable paradox wherein Group 2 (CCR 80–120 mL/min) showed a 51% lower hazard of mortality and Group 3 (CCR > 120 mL/min) demonstrated an 87% reduction in mortality risk compared to Group 1. While literature suggests that CCR > 120 mL/min may indicate hyperfiltration, particularly in individuals with diabetes or obesity—conditions associated with increased mortality—our study highlights a different perspective. We adjusted for diabetes as a confounding factor in our analysis, recognizing its significant impact on kidney function and mortality risk (52). This adjustment indicates that the relationship between CCR and mortality exemplified in our findings reflects the severity of renal impairment in patients with lower CCR rather than merely the hyperfiltration effects associated with diabetes. Additionally, our single preoperative measurement of CCR may not fully capture the chronic renal adaptations occurring over time in patients with these conditions. Chronic conditions such as diabetes lead to renal adaptations over time, including periods of increased filtration (hyperfiltration) (53, 54). Therefore, future research should explore longitudinal changes in CCR to better understand its correlation with mortality risk within these populations. Furthermore, although hyperfiltration may suggest an elevated risk in specific cohorts, our results imply that higher CCR levels, within certain ranges, are associated with improved survival outcomes in the context of our study. This reinforces the potential for regular renal health monitoring to guide clinical management and intervention strategies for patients at risk.

This study presents several significant advantages, including an adequately large sample size and an extended follow-up period, both of which substantially enhance the reliability and robustness of our results and effectively represent the older demographics of Chinese individuals with OPFs, which strengthens the external validity of the results. Moreover, the open enrollment methodology reduces the potential selection bias and ensures a diverse participant population. The extended follow-up period enabled a comprehensive analysis of mortality across varying levels of CCR, yielding valuable insights into long-term outcomes within this high-risk group.

Nonetheless, certain limitations of this study warrant consideration. While mortality served as the primary endpoint because of its objective nature and clinical relevance, this singular focus may have overlooked other important clinical outcomes such as quality of life, hospitalization rates, and functional status. In addition, the exclusive use of the Cockcroft–Gault formula for CCR estimation, although widely accepted, may not fully capture renal function compared to other methods such as CKD-EPI or MDRD equations. Additionally, cohort studies inherently possess limitations, including potential biases in participant selection, confounding variables, and challenges in establishing causality owing to their observational nature. Despite robust statistical adjustments, residual confounding factors could not be completely ruled out. Furthermore, the single-center design and predominantly homogeneous patient population may have restricted the external validity of our findings across different healthcare settings and ethnic groups. To enhance the generalizability and robustness of these results, future studies should use multicenter randomized controlled trials with standardized protocols that incorporate diverse ethnic populations and multiple CCR estimation methods.

## 5 Conclusion

In conclusion, this study provides compelling evidence of a negative relationship between CCR and mortality in hospitalized patients with OPFs. These findings strongly indicate that higher CCR levels are associated with a lower risk of mortality, highlighting the importance of evaluating renal function in this patient population. Moreover, this association remained robust even after adjusting for multiple confounding factors. Given that low CCR levels may represent a modifiable risk factor, monitoring and improving renal function in these patients are critical for enhancing their overall prognosis. The implementation of early screening protocols and targeted interventions in patients with reduced CCR may help improve clinical outcomes. The substantial sample size extended the follow-up period, and comprehensive statistical analyses supported the reliability of these findings. However, future multicenter studies are warranted to further validate these results, expand their applicability across diverse populations, and investigate potential therapeutic strategies for CCR improvement.

## Data Availability

The raw data supporting the conclusions of this article will be made available by the authors, without undue reservation.
