# Supplementary material for: Association between creatinine clearance and mortality in Chinese patients with osteoporotic fractures: a retrospective cohort study
Source: Front Med (Lausanne). 2025 Aug 14;12:1550525. doi: 10.3389/fmed.2025.1550525 (PMC12392318; doi:10.3389/fmed.2025.1550525)
Supplement: Supplementary file 1 [file Table_1.doc]

**Table S1.** Subgroup analysis between CCR and mortality.

|  | N | Mortality^a^  HR (95% CI) *P*-value |
| --- | --- | --- |
| PT, s |  |  |
| low | 1529 | NA |
| high | 1611 | 0.98 (0.97, 0.99) <0.01 |
| APTT, s |  |  |
| low | 1569 | 0.98 (0.97, 0.99) <0.01 |
| high | 1570 | NA |
| Platelet, ×10^9^/L |  |  |
| low | 1571 | 0.98 (0.97, 0.99) <0.01 |
| high | 1594 | NA |
| Hemoglobin, g/L |  |  |
| low | 1547 | 0.97 (0.97, 0.98) <0.01 |
| high | 1618 | NA |
| Albumin, g/L |  |  |
| low | 1544 | 0.97 (0.97, 0.98) <0.01 |
| high | 1582 | 0.98 (0.97, 0.99) <0.01 |
| Calcium, mmol/L |  |  |
| low | 1552 | 0.97 (0.96, 0.98) <0.01 |
| high | 1622 | 0.98 (0.97, 0.99) <0.01 |
| Neutrophil, ×10^9^/L |  |  |
| low | 1548 | NA |
| high | 1617 | 0.98 (0.97, 0.99) <0.01 |
| Lymphocyte, ×10^9^/L |  |  |
| low | 1389 | 0.98 (0.97, 0.99) <0.01 |
| high | 1776 | NA |
| Monocyte, ×10^9^/L |  |  |
| low | 1556 | 0.98 (0.97, 0.99) <0.01 |
| high | 1609 | 0.98 (0.97, 0.99) <0.01 |
| Potassium, mmol/L |  |  |
| low | 1580 | 0.98 (0.97, 0.99) <0.01 |
| high | 1594 | 0.98 (0.97, 0.99) <0.01 |
| Uric acid, μmol/L |  |  |
| low | 1581 | 0.98 (0.97, 0.98) <0.01 |
| high | 1594 | 0.98 (0.97, 0.99) <0.01 |
| ASA, N (%) |  |  |
| 1 | 467 | NA |
| 2 | 2083 | 0.98 (0.97, 0.99) <0.01 |
| ≥3 | 627 | 0.98 (0.97, 0.99) <0.01 |
| Hypertension, N (%) |  |  |
| no | 2787 | 0.98 (0.97, 0.98) <0.01 |
| yes | 390 | 0.98 (0.96, 0.99) <0.01 |
| Diabetes, N (%) |  |  |
| no | 3069 | 0.98 (0.97, 0.98) <0.01 |
| yes | 108 | NA |
| Tumor, N (%) |  |  |
| no | 3143 | 0.98 (0.97, 0.98) <0.01 |
| yes | 34 | NA |
| Shock, N (%) |  |  |
| no | 3175 | 0.98 (0.97, 0.98) <0.01 |
| yes | 2 | NA |
| Smoking, N (%) |  |  |
| no | 2769 | NA |
| yes | 253 | 0.25 (0.00, 961.36) 0.74 |

^a^Patients were stratified based on PT, APTT, platelet, hemoglobin, albumin, calcium, neutrophils, lymphocytes, monocytes, potassium, uric acid, ASA, hypertension, diabetes, tumor, shock, and smoking, and additional covariates not included in the stratification were adjusted for in the analysis.
